# Psychosocial Influence of Ehlers–Danlos Syndrome in Daily Life of Patients: A Qualitative Study

**DOI:** 10.3390/ijerph17176425

**Published:** 2020-09-03

**Authors:** Inmaculada C. Palomo-Toucedo, Fatima Leon-Larios, María Reina-Bueno, María del Carmen Vázquez-Bautista, Pedro V. Munuera-Martínez, Gabriel Domínguez-Maldonado

**Affiliations:** 1 Department of Podiatry, University of Seville, 41009 Seville, Spain; ipalomo@us.es (I.C.P.-T.); carmenvaz@us.es (M.d.C.V.-B.); pmunuera@us.es (P.V.M.-M.); gdominguez@us.es (G.D.-M.); 2 Department of Nursing, University of Seville, 41009 Seville, Spain; fatimaleon@us.es

**Keywords:** Ehlers–Danlos syndrome, qualitative study, life experiences, psychosocial approach, chronic pain

## Abstract

(1) Background: Ehlers–Danlos syndrome is a heterogeneous group of connective tissue disorders causing pain, fatigue, and disabilities; it has several implications for patients who suffer from this disease. The major clinical manifestations of EDS include joint hypermobility, skin hyperextensibility, and generalized connective tissue fragility. This research aims to explore their perceptions and experiences about the phycological and social spheres. (2) Methods: Semistructured interviews were carried out. Participants were encouraged to talk about issues related to their disease by asking open-ended questions in one to one interview. The interview guide included questions to identify the syndrome’s influence on the social and psychological life of patients All interviews were audio recorded, fully transcribed, and analyzed using the phenomenological theoretical framework. The method of analysis was the thematic interpreting of perspectives and approaches. (3) Results: 31 individuals were proposed to participate in this study. Five patients refused to participate, so a total of 26 interviews were performed. Six themes ((1) Pain and its consequences on a daily basis; (2) The need to name the problem: the diagnosis; (3) Restructuring leisure and social relationships; (4) Limitations due to economic conditions; (5) Psychological impact of the disease situation; (6) Professional limitations) and four subthemes ((1) The value of partner support; (2) The weather influence on social plans; (3) Physical exercise and illness; (4) Support groups) emerged from the data. (4) Conclusions: This study revealed the impact of the syndrome on the social and daily life of patients, and not only in a physical level, but also in a psychological and social approach. These findings allow healthcare providers to know more about this disease in order to support and give advice to patients about the changes they will have to make.

## 1. Introduction

Ehlers–Danlos syndrome (EDS) is a heritable disorder of connective tissue with a prevalence of 1:5000 and 1:20,000 around the world, being more frequent in females, who represent 90% of all cases [1,2].

Although symptoms arise at childhood, they become more severe in adulthood. The major clinical manifestations of EDS include joint hypermobility, skin hyperextensibility, and generalized connective tissue fragility [3,4]. Their main clinical manifestations are related to multiple body systems expressing complex and diverse symptoms, being chronic pain and fatigue the most frequent ones [1] similar to symptoms present in other connective tissue disorders, which makes the specific diagnosis difficult [5]. One of the major clinical manifestations in patients with EDS is pain, which may be associated with disability, depression, anxiety, or physical and mental health-related quality of life impairment [6]. Fatigue has also been identified as one of the most debilitating symptoms in EDS [7], and it is highly related to sleep disturbances, concentration problems, poor social functioning, and pain severity [8].

In a study carried out in 2018, in which 80 patients diagnosed with EDS participated, 67.1% reported a prevalence of habitual pain and 68.8% reported fatigue [9]. Chronic pain is a manifestation of Ehlers–Danlos syndrome that has impact on the patients’ physical, social, and psychological health. Although physical implications have been widely described, researchers have explored less the psychological and social implications and little is known. These aspects also have an impact on the patients’ quality of life, so it is interesting to investigate how patients who suffer from this disease face their daily life, and its influence on their psychosocial life.

Previous studies have shown that the lack of knowledge about EDS among health professionals is usual [9]. The experience of living with a rare genetic disease is complex, and the individual’s life might be affected. Although a biomedical model is common, a biopsychosocial approach should be assumed. A way of improving the quality of life of these patients is the development of advancements in medical treatment, but also with interventions that contribute to get better psychosocial factors that may have an influence on their daily life [10]. More research is needed to explore psychological and social impact of living with a rare disease in order to identify potential risk factors give information to health professionals, so that the patient´s quality of life can be improved [11,12].

The aim of this paper is to explore the experiences and feelings of those patients diagnosed with EDS and how this influences their daily life. In addition, this information helps healthcare professionals to advise them to adapt to their new circumstances by providing support and treating them as a whole.

## 2. Materials and Methods

This research utilized qualitative methods employing thematic analysis by Clarke and Braun (2006) [10]. Individual semistructured and in-depth interviews were conducted between April 2018 and September 2019 in four locations in Spain (A Coruña, Seville, Jaen and Barcelona). The study adhered to the COREQ (COnsolidated criteria for REporting Qualitative research); a 32-item checklist using in qualitative studies [11].

A purposive sampling strategy was followed. Participants were recruited from different patient associations in Spain, subsidiaries of the National Association of Ehlers–Danlos Syndrome, Hypermobility and Collagen Disease (ANSEDH), after asking for collaboration. To preserve privacy, interested members telephoned a designated investigator directly for further information. According to the geographical dispersion, we received patients in various public and private centers, in the four cities cited before. Inclusion criteria for participants were 18 years of age or over, with a diagnosis of EDS, and the ability to speak Spanish. None of the participating women were pregnant. A photocopy of the clinical report with molecular or clinical confirmation of EDS was recorded and the subtype, usually established by a physician from a rheumatology service. All of those affected were classified either as classical EDS type I, classical-like EDS type II, or hypermobile EDS type III (*n* = 25), except a woman, P18, with vascular EDS type IV diagnosis. Conditions such as age, gender, employment status, and geographical location were taken into account in order to have a composite sample. Later, a member of the research group contacted all participants in order to arrange the place and time of the interview. A friendly style of conversation and was used, so that the anxiety of participants was reduced. A wide, comfortable room with pleasant atmosphere and plenty of time for each meeting were provided.

### 2.1. Data Collection

Participants were encouraged to talk about issues related to their disease by asking open-ended questions in one to one interview. The interview guide included questions to identify the syndrome’s influence on the social and psychological life of patients (Table 1).

The development of these questions was based on previous literature [6,12,13,14,15]. The participant was alone with the investigators. The research team used open questions in order to allow participants to share their experiences and feelings widely. These questions were pilot-tested prior to study and agreed upon by the team.

All interviews were audio recorded in Spanish, fully transcribed, and revised for accuracy. Besides, all information was compared and contrasted with investigators’ notes. Two members of the research group with extensive experience in in rheumatologic diseases and quality of life research, both a man and a woman so that participants of both genders sexes felt comfortable, always carried out the interviews. Interobserver variability was controlled because both of them received a previous training to conduct the interview properly. Interviewers did not have a professional or private relationship with any of the patients. The duration of the interviews was on average 41 min (range 27 to 55) included in this paper were translated into English for this paper by a native English speaker and subsequently translated into Spanish again, in order to ensure the accuracy of the translation. Any inconsistences identified were resolved by going back to the recorded audio. The final themes were consensual among researchers.

### 2.2. Data Analysis

One of the authors, experienced in qualitative research, analyzed the transcripts. The rest of the research team read all the transcriptions and agreed on the meaning of some ideas expressed by participants. The thematic analysis of Braun and Clarke (2006) [10] was carried out using a qualitative software package NVivo 1.0 to analyze data (QSR International, Melbourne, Australia). Thematic analysis emphasizes identifying, analyzing, and interpreting patterns of “themes” within qualitative data [16]. Emerged themes and subthemes were discussed with an inductive approach, deriving from the data. There was agreement among the research team about the themes and subthemes identified. This method allows us to identify, analyze, report, and understand the meanings and experiences disclosed by patients with EDS from their own perspective. The first step in this process was to read all interviews and understand all data. After this, preliminary codes were assigned to data in order to describe the content. Then, all the statements were classified according to themes and subthemes anchored on textual narratives intensively and repetitively; the main aim was to search for patterns and interconnections. The emerging themes were included and compared with the data. After this step, the research team involved in the study agreed upon the drawn up findings [17].

Reflectivity was maintained throughout the study by controlling previous assumptions and held beliefs through reflection notes. Theming was influenced by the data and there was minimal interpretation. Rigor and trustworthiness was guaranteed through the process of analysis. Themes were reviewed among authors and resonated with patients and were consistent with their experiences.

### 2.3. Ethical Considerations

Written consent was obtained from all the participants first. Later, the verbal consent for audio recording the interview was made at the beginning of the performance, identifying themselves with their full name. This study was authorized by the Research Ethical Committee of Virgen Macarena and Virgen del Rocío Hospitals with number 1754-N-17 and obtained a favorable evaluation. This study complied with the legislation on personal data protection, and the ethical principles of the World Medical Association Declaration of Helsinki were followed [18]. Confidentiality was guaranteed to all participants. A code was assigned to every single participant. Interviewers were not involved in the participants’ medical process. Participants were informed about the objectives and procedure of the study. This research was not funded by any specific organizations or funds.

## 3. Results

Thirty-one individuals were proposed to participate in this study. A total of five patients refused to participate mainly due to lack of availability to answer the interview. The reported data were collected from 26 individual interviews reaching saturation of information. Participants´ sociodemographic characteristics are detailed in Table 2.

Verbatim testimonies are provided to illustrate the findings. The analysis provided six key interrelated themes and four subthemes presented in Table 3.

The main themes identified from the analyses were (1) pain and its consequences on a daily basis, (2) the need to name the problem: the diagnosis, (3) restructuring leisure and social relationships, (4) limitations due to economic conditions, (5) psychological impact of the disease situation, and (6) professional limitations. The subthemes that emerged from each theme are outlined for each theme.

### 3.1. Pain and Its Consequences on a Daily Basis

One of the most common and frequent manifestations is pain. Many interviewees state that they have learned to live with chronic pain and that is not a problem for them anymore.

*“… in the end, you get used to pain …”.*

*(P1)*

However, what limits them the most is fatigue and exhaustion derived from the lack of rest at night, becoming a restriction for the performance of daily routines.

*“Since I don’t sleep and I don’t rest well, I don’t have a comforting sleep … Tiredness is worse than pain”.*

*(P2)*

*“… What limits me the most is fatigue, physical fatigue. Because you are … You wake up tired”.*

*(P3)*

*“Fatigue is much worse than pain, fatigue conditions a lot of things”.*

*(P8)*

### 3.2. The Need to Name the Problem: The Diagnosis

Patients relate their journey until they are finally diagnosed. In some cases, they spend many years until a diagnostic label is assigned to all the symptomatology they express. This is motivated to a great extent by the fact that their physical appearance is completely normal, and sometimes they feel misunderstood and judged by health professionals and their own family and friends.

*“You go a long road until you get a diagnosis”.*

*(P1)*

*“The diagnosis thing is something you wait for with your arms wide open, because people see you as a hypochondriac… And you feel bad, because nobody, no one believes you, because apart from pain, there is physical exhaustion, which I personally believe is the worst thing I have to deal with, not having energy for anything”.*

*(P10)*

*“Great, I have something, I’m not crazy!”.*

*(P13)*

However, patients express the need of labeling what is happening to them, in order to cope more effectively with the disease’s development.

*“A lot of times, they ask you “Why do you want to put a label on it?”. Yes, you do need to put a label and know what you’re facing, how to prevent it and how to improve”.*

*(P2)*

*“… It is not encouraging or positive, it really is a necessary part so that you can take the time you need in order to accept that this thing is going to be there with you forever. And from there, you can adapt your life to that fact. It’s not the most important thing for me, but it really is necessary (referring to the diagnosis)”.*

*(P22)*

On the other hand, some patients express a change in attitude from health professionals once diagnosis is made. From that moment, they feel less judged and more supported in the process.

*“Going to a doctor and not being looked at like a weirdo, not seeing the doctor’s face being like “ My dear, what a pain in the neck’’, not feeling that he thinks you’re a hypochondriac, or that you’re trying to get something. Feeling understood, precisely, listened to, valued, feeling that you’re … that you’re supported, that they are telling you ‘’don’t worry, we’ll do everything we can possibly do’’. Believing, even when it’s not true, that you are protected and that they’re helping you”.*

*(P10)*

### 3.3. Restructuring Leisure and Social Relationships

Generally, people who suffer from EDS relate how they have to deal with the social image that people around them have, as, although they have a normal appearance, they have many restrictions on the performance of their daily routines. They are sometimes branded as lazy, apathetic, and tired people who cannot undertake a project, when actually they are too exhausted to do so.

*“… People around you don’t quite understand you neither, see? They see you’re okay, you’re happy, you work a lot of hours, whatever, but of course, then they don’t understand that when you reach home you are incapable …”.*

*(P1)*

*“… And they think that I’m home because I want to be. No, that’s because I can’t walk on the street, because I’m going to fall, or because it hurts me so much that I choose not to walk”.*

*(P6)*

*“… There are people who understand you and well, they give you some support, and there are people who don’t, they don’t understand it because hey, I am a normal person on the surface, I mean, no one would say at first sight that I have a chronic disease, but of course I can’t prove that”.*

*(P7)*

*“Usually, there is nowhere to sit on the train (…). And since people see that you’re fine, obviously no one gives you their seat”.*

*(P13)*

It is possible to see social isolation behaviors in some patients, as they are unable to follow the pace that some groups have, as well as to make short-term social plans, because they cannot know how are they going to feel.

*“… It is true that you start to lock yourself up more and more, you see people less, you find more obstacles for everything …”.*

*(P2)*

*“It does affect me more in the social aspect, and if I have to be in a bar, sitting on a chair, with a backache or whatever, and loud music, and in the night, not seeing well, forcing my eyes … Then I think “Look, I’d rather stay in bed, that’s it””.*

*(P5)*

*“There are people who don’t call me, who don’t count me in anymore”.*

*(P6)*

*“… Social life deteriorates a lot, because a group is very dynamic, and the group keeps on moving either you can or can’t go out (…) we are people who try to keep with our normal life and maintain our daily habits, but well, there are moments in which you have limitations and not everyone understands it”.*

*(P22)*

To do so, some of them have the strategy of resting more on the previous and subsequent days, so that they can make those social plans.

*“If I want to go out, I have to rest more, I have to go with a process … I go out with more programming”.*

*(P8)*

All of this adds difficulty to the patients’ social relationships, up to the point that they have to justify themselves in front of the others, as they do not always feel understood in their acts and behavior.

*“… I say ‘’I’m busy, I’m very busy’’. It’s not true that I have a lot of things to do, what happens is that I take so much time in every little thing that I do, it is so difficult to me, that in the end I don’t have any more time”.*

*(P2)*

*“So you can go out for some beers and you can’t clean your house?” (…) “Well, yes, turns out I can’t clean the house but I can go out and have a beer, because lifting up the glass is not the same effort as putting around the feather duster, it’s not the same thing, is it?”.*

*(P13)*

*“I don’t go out. Because I can’t stand up for too long and sure I can’t have long walks, because I need to be making stops all the time. I just can’t, my knees get weak, they hurt”.*

*(P17)*

#### 3.3.1. The Value of Partner Support (Subtheme)

It is fundamental that the patient’s partner understands the disease, so that they know about certain situations that can happen. If this is not the case, problems in the couple are usual.

*“So your social life is quite complicated and the same thing with relationships. I had a boyfriend and he used to squeeze me, and I had bruises all over me, and he used to say ‘’it’s like you were going to break, don’t you realize I’m not doing anything? It is you’’. And I was 18. So … You can’t go into a relationship either, because men don’t understand your disease, or they think you’re lazy, or …”.*

*(P5)*

*“Sometimes, only sometimes they do understand you, and sometimes they don’t (referring to the partner)”.*

*(P14)*

*“That has an effect on the relationship, because the main thing is that your partner has to know what you’re going through, and they have to learn how to accept things, and each person has a different rate when it comes to accepting things”.*

*(P22)*

Especially in the case of women, problems related to the sexual sphere have been identified. Many times, their partners do not believe they really have a health problem; instead, they think it might be that they complain a lot.

*“… It is difficult that anybody understands you, because what they think is that you’re making excuses. It is very complicated. At a given time, you have pain, you look for a different position, you change, but the fact of being exhausted and thinking ‘’my God, how lazy, now this and that, and whew’’… That is tremendous”.*

*(P10)*

*“I mean, even if he doesn’t say it, I think it is so, that since I was given the diagnosis he thinks there’s a reason. Before, he used to think it was due to … arthrosis, due to work … right? And yes, I think so, that now he is more like … more positive, you know? As if he was thinking ‘wow, if she has this thing, I have to bear with her’”.*

*(P17)*

#### 3.3.2. The Weather Influence on Social Plans (Subtheme)

Atmospheric changes have an impact on the physical conditions of people with EDS. Humidity and pressure changes seem to have a negative effect, and when this happens, patients feel obliged to modify their social plans.

*“When there’s nice weather I can do more things, I mean, it’s easier to do them, but actually, I always have limitations”.*

*(P1)*

*“It’s true that when it’s colder I have more contractures and more pain”.*

*(P4)*

*“Any contrast in temperature affects me greatly. Is it going to rain? Then don’t count on me for the next three days”.*

*(P9)*

#### 3.3.3. Physical Exercise and Illness (Subtheme)

There are patients that used to have a very active life until diagnosis; some of them even used to compete professionally, but over the years and with the disease’s development, physical exercise became harder and harder. There are some who have abandoned it due to the difficulty of performing it.

*“… Because I used to walk every day, but I had to give it up due to physical problems. My hip used to hurt me a lot”.*

*(P2)*

*“Before the hardest part of this, I used to do potholing, I’ve been in a federation for 16 years, I worked as a monitor, I used to do diving, rafting, ice-climbing …”.*

*(P21)*

Some of them keep on doing physical exercise, and the activities they tolerate the best are walking, swimming, yoga, and Pilates. They express they sense a disease improvement with these activities.

*“And doing gymnastics has helped me, because it relieved my backaches. Pilates was good for me, soft gymnastics, stretching exercises …”.*

*(P5)*

### 3.4. Limitations Due to Economic Conditions

Differences can be observed in the way people handle the disease depending on their purchasing power and economic conditions. The Spanish health service has a coverage treatment, but it does not have other treatments that contribute to a symptom’s improvement. These are not accessible to everybody, so there is inequality.

*“Medication is quite expensive … I can’t even consider signing up, because I can’t pay the swimming pool and yoga as well”.*

*(P3)*

*“What I do is walk, stroll, I’ve been in a gym but I had to give it up, because I can’t afford it (…) I would love to go to physiotherapy, especially because of the backaches, I would really love to, to get some massages on my legs, reflex therapy, everything, everything that can cure my pain. I would love to go, but I can’t afford it”.*

*(P12)*

### 3.5. Psychological Impact of the Disease Situation

People who suffer from EDS confirm the negative impact the disease has on their psychological well-being and balance, and still, they do not feel the psychological or emotional support that would help them cope with the disease’s effects.

*“We get depressed because we have pain, not the other way around … Chronic fatigue is not about resting, you already wake up tired”.*

*(P6)*

*“The truth is, it’s hard. It’s hard because it affects a lot, psychologically, just thinking that … You feel useless, totally useless, unable, it’s a continuous fight between what your head wants to do, what your mind asks you to do, and what your body allows you to do, and it’s impossible. And you can’t control … This is very hard to deal with, psychologically. It’s very hard, the disease itself is very hard”.*

*(P10)*

*“And sure, it affects me psychologically, because I would like to do tons of things, such as hiking, and I can’t. I can’t go and make an excursion, just like that; the next day I would be 2 days lying in bed without being able to get up”.*

*(P12)*

#### Support Groups (Subtheme)

Support groups, patient associations, etc. bring positively valued support in a first instance, as they help patients be aware of the fact that they are not alone and that there are more people who share their same problems.

*“In the beginning, you … You feel the need of seeing and knowing people like you. It’s a very big help, getting to talk about these things with people like you”.*

*(P2)*

*“It brings more consolation, because then you see that it doesn’t happen only to you, or you see that you’re not exaggerating, that there are others who experience it the same way as you do”.*

*(P3)*

However, after a time, they do not find so much usefulness anymore, and participants communicate that they would rather not share these spaces with other people.

*“There are times that you get tired of doing always the same thing and you can also feel a bit weighed down”.*

*(P2)*

*“Hearing all these issues that other people have gives me such anxiety that I can’t even speak about it”.*

*(P12)*

*“There are people who experience this association thing as something positive, and there are others who say that, well, that it’s more like a burden for them”.*

*(P14)*

### 3.6. Professional Limitations

Patients tell their stories about how their disease restricted them more and more in the professional aspect, which sometimes led them to apply for work leave for several seasons during their professional life. This has occasionally been the reason for some of them being fired, due to their supervisors’ lack of understanding regarding the peculiarities of the disease’s symptomatology. Nevertheless, when a work change is possible, adapted to each person’s abilities, they feel satisfaction with their performance at work.

*“And yes, I had to change my job because I couldn’t bear it (…) On one hand, the syndrome wouldn’t allow you do the job, but on the other hand they don’t wanna tell you that you have some disability”.*

*(P1)*

*“Terrible, I mean, in the professional aspect, very bad. The moment they know that you have some disease or when you start having leaves more frequently, we all know what’s coming …” (referring to work redundancy).*

*(P7)*

In other cases, patients had to adapt their professional life to the limitations caused by the disease, reducing the number of hours or the type of work they performed.

*“Luckily, I was in a very flexible firm until now, so I could decide not to go some days, or make up for those hours some other day; I could clock in later as I arrived and as I left, I could manage my schedule the way I preferred to”.*

*(P18)*

*“And at a work level, that things got a little facilitated, because sometimes you’re not as if you needed to ask for disability, but you’re neither at your 100% and that’s a shame, because I would like to keep on working”.*

*(P20)*

## 4. Discussion

The aim of this research was to gain more information about the impact of Ehlers–Danlos syndrome on the psychosocial life of those diagnosed. The results of this study reveal that this disease has a wide and deep impact on patient’s psychosocial life, as the numerous themes identified suggest. This information pretends to cover the lack of knowledge in the health care system about this rare disease referred by patients, by discussing, providing, and sharing experiences and their consequences in daily life.

Only two recent studies related to this field have been published [19,20]. These authors conducted an evaluation through a qualitative study based on patient interviews. Qualitative studies are common and useful for understanding the feelings and experiences that underlie health behaviors, so this kind of research is the most common for covering these aspects [21,22].

As Bennett et al. (2019) [19] reported, patients suffering from EDS stated an overall lack of awareness of this disorder among healthcare professionals, but also among the general public [23]. Our research claims to shed light on how the syndrome affects the psychological and social lives of patients. This aims to help healthcare providers advise patients on how their lives will change after the diagnosis and how can they adapt to their new circumstances providing counseling and support but empowering patients and treating them as a whole.

Some studies suggested that the main problems caused by EDS are chronic pain and fatigue, as our interviewees referred [1,12,24,25]. These symptoms have a high impact on daily activities, up to 87% patients feel pain, according to Voermans et al. (2010) [7], and this contributes that patients do not feel the energy to perform activities in a normal day. Moreover, the pain experience may be exacerbated by psychological and emotional problems, such as depression, anxiety, affective disorder, lack of self-confidence, negative thinking, hopelessness, or desperation [26]. On the other hand, this limitation to follow a daily routine has an impact on their social life, even contributing to isolation [19,20,27]. We observed that our participants referred they are used to avoid social situations quite often because of discomfort suffered. As other research has shown, clinical manifestations of EDS are associated with the social isolation of patients. Many people often have to give up on activities and put restrictions to family and friends due to their fear of pain or dislocations of joints, fear of falling due to their impaired balance, or fear of movement, which contribute to their isolation [13,19,20,25,28,29,30]. It is difficult for them to have a regular social life because that means complaints in the body in the following days. It is necessary to balance activity and rest, organizing activities according to personal level of fatigue and pain [27].

A published systematic review detected the absence of good clinical practice guidelines to optimize EDS patient care; in fact, many patients remain without a correct diagnosis [19,20]. Many of them have symptoms and do not meet the criteria to be diagnosed at the moment, and this lack of medical knowledge contributes to the patient’s burden and to a delay in diagnosis and treatment. Baeza-Velasco et al. (2018) [9] detected a diagnosis delay of 22 years on average in the participants of their study. As De Baets et al. (2017) [27] found, our study has shown that people with a diagnosis perceived this as a need to get social credibility. Patients with EDS diagnostic feel that, once they know their disease, they can handle the process and prevent complications better. However, the path to reach this diagnosis is often defined as a very frustrating, challenging, and slow process [19,20,27]. As they sometimes do not have observable physical signs, people with Ehlers–Danlos syndrome do not feel the understanding of care providers. This is a common inconvenience of EDS, also assumed by participants in our study. The survey respondents of Arthur et al.’s study (2016) [6] indicated the need to seek out for diagnosis confirmation with multiple physicians, which implied the difficulty these patients faced when trying to access the appropriate treatment. Patients in our study manifested that this situation changed after diagnosis, although not always. Some healthcare providers do not know the implications of this disease, and patients feel invisible and often struggle to find a professional with whom to discuss what they feel [14]. Those are the reasons why it is important to disseminate knowledge about EDS in order to minimize negative these health consequences, being a challenge for nurses who care and support these patients.

The results about employment found in our study are in line with previous studies that have proved that the limitations of individuals with EDS decrease career opportunities and determine what kind of activities they are able or not to do at work. Berglund & Nordström (2001) [31] observed that EDS patients had a poorer self-rated functional health status than fibromyalgia and rheumatoid arthritis patients, even in work-related dimensions. Some patients are forced to quit their jobs due to symptoms as chronic pain [32]; others have to reduce their work hours or switch their professional activities to a different one, better adapted to their abilities [27,33]. Support groups and patient groups have been proven to help reduce fear and anxiety related to symptoms [34]. Our participants highlighted the help received in these groups when it comes to facing the diagnosis of the disease in the first stages. Interventions that give the patients knowledge and tools to manage activity limitations and pain in their daily life, through raising awareness of thoughts and patterns in life (cognitive-behavioral treatments), have proven to be effective [35] and useful to improve patient’s ability to handle experiences associated with chronic pain [36].

Our findings confirm previous evidence on how patients’ social life had an impact on activities or ways of relating. This invites to adapt hobbies and leisure time according to the new situation. Subjects interviewed by Bennet et al. (2019) [19] described limitations in daily activities due to factors such as frequent dislocations, restricted mobility and symptoms of fatigue and pain. Other authors have previously related fatigue severity to low activity participation [37] as we found in our research, participants referred that fatigue after a restless night was the main reason to refuse plans the day after. Scheper et al. (2017) [38] observed that patients with hypermobility type of EDS showed less muscle strength, and this was associated with activity limitations. Our participants mentioned depression in subjects suffering from benign joint hypermobility syndrome, and they confirmed that they chose plans to join depending on their capacity affecting severely their social life. They did not do what they liked but what they could do. In this way, their physical limitations determined their social life.

Exercise and physiotherapy are an important part of patient management for some authors [33], providing functional positive effects [29,30] and even contributing to improve their mental health [39]. On the other hand, a systematic review carried out by Palmer et al. (2014) [40] concluded that a clear cause–effect relationship between exercise and improvement of symptoms in patients with EDS type hypermobile and joint hypermobility syndrome was not demonstrated. However, the Spanish health service does not always cover this treatment. This is a comparative grievance between people who can defray expenses and those who cannot, contributing to inequality according to socioeconomic criteria. There is a clear need for health professionals to receive training and knowledge in order to provide biopsychosocial support for people with this condition, as patients often perceived to be poorly understood by healthcare providers [41]. In this way, patients will be able to adapt better to their disease.

Smith et al. (2014) [42] found a fourfold increased risk of anxiety and depression in EDS patients. Berglund et al. (2015) [14] observed that 74.8% of the participants with EDS reported high anxiety scores, and probable depression was rated by 22.4% of them. Albayrak et al. (2015) [43] reported a significantly increased level of depression in the participants of their study with EDS. This psychological involvement has been identified in our interviewees who refer psychological symptoms associated with chronic pain and fatigue. This experience of pain, especially if chronic, can contribute to these negative emotions [12].

Female participants of this research showed difficulties to fully enjoy their sexual life. In some cases, they did not feel understood by their partners. The most common problems were related to positions during intercourse and lack of desire, due to pain and fatigue, in some cases. Many pelvic problems have been related to multiple dysfunctions in EDS patients [44], such as pelvic, rectal, or genital prolapses; vaginal dryness; and other gynecological and musculoskeletal features that may constitute common forms of pelvic pain. Some participants interviewed by Bennett et al. (2019) [19] also reported difficulties with sex and intimate relationships. These problems seemed to stem from issues around fatigue and pain as we found and other authors stated [32].

Limitations: In addition, all participants were members of a patient association, so their experiences or perceptions about their illness may have been changed along time or distant from those without such social support. Furthermore, due to the number of women affected, the differences between genders cannot be assessed. Despite the lack of ethnic diversity in the sample of this study, considered a limitation, this paper contributes to have a more psychosocial approach and to add the need of focusing on the patients’ adaption of processes, so that they can have a good quality of life.

Further researches are needed from quantitative and qualitative approach. Because pain and fatigue are common, the relationship with fibromyalgia in adults and false growing pains in children should be identified, for early diagnosis and better social and health professional comprehension of the disease.

On the other hand, how those symptoms can be managed by patients and how they might affect their emotional and social health must be explored.

Results provided by this paper are useful to design interventions that support patients in their psychosocial, disease-related problems. Healthcare professionals will be the key, helping patients reorganize their lives according to the specific symptoms of the disease and giving them advice to get a better daily quality of life and manage the disease, besides medical treatment. Physicians, nurses, occupational therapists, social workers, and psychologists, among others, should be part of the multidisciplinary team necessary to achieve this goal and manage the disease.

## 5. Conclusions

This study suggests that EDS affects daily life, not only in a physical level, but also in a psychological and social sense. Social and familiar support is important for patients in order to face the syndrome. Patients have to be aware of the changes in their social life because of the disease so that they can have a better quality of life. Their professional opportunities are reduced by the progression of symptoms and economic restrictions may diminish their self-care. Healthcare providers involved in the care of patients with EDS need to know it and integrate this information in order to provide a better care to patients who want to improve their quality of life.

## Figures and Tables

**Table 1:** Interview guide.

1. *Which physical affections do you think this disease has on your health?*
2. *Which psychosocial aspects of your daily life are affected by the disease?*
3. *Which aspects/situations/factors do help you cope better with the disease?*
4. *What do you think that determines the disease severity and the impact it has on your daily life?*

**Table 2:** Participants’ sociodemographic characteristics.

Participant	Age	Gender	Education	Occupation
P1	42	Male	Primary	Autonomous assembler
P2	46	Female	University	Engineer
P3	38	Female	Primary	Caregiver
P4	18	Male	Secondary	Student
P5	49	Female	Secondary	Administrative clerk
P6	47	Female	University	Executive
P7	37	Female	Primary	Hairdresser
P8	48	Female	Secondary	Farmer
P9	51	Female	Secondary	Shop assistant
P10	53	Female	Secondary	Administrative clerk
P11	25	Male	Secondary	Student
P12	54	Female	Secondary	Occupational therapist
P13	41	Female	Secondary	Administrative clerk
P14	42	Male	University	Pharmacist
P15	32	Female	Secondary	Marketing
P16	57	Female	Secondary	Civil servant
P17	56	Female	Primary	Fishmonger
P18	26	Female	University	Computing
P19	46	Female	University	Biologist
P20	33	Female	Secondary	Marketing
P21	48	Female	University	Administrative clerk
P22	40	Female	University	Teacher
P23	45	Female	University	Bank clerk
P24	24	Male	Secondary	Student
P25	20	Female	Secondary	Student
P26	57	Female	Secondary	Nursing assistant

**Table 3:** Themes and subthemes.

*Main Themes*
1. *Pain and its consequences on a daily basis*
2. *The need to name the problem: the diagnosis*
3. *Restructuring leisure and social relationships*
* Subthemes*
a. *The value of partner support*
b. *The weather influence on social plans*
c. *Physical exercise and illness*
4. *Limitations due to economic conditions*
5. *Psychological impact of the disease situation*
* Subthemes*
a. *Support groups*
6. *Professional limitations*
